# Supplementary material for: Dynamic transcriptomic profiles of zebrafish gills in response to zinc supplementation
Source: BMC Genomics. 2010 Oct 11;11:553. doi: 10.1186/1471-2164-11-553 (PMC3091702; doi:10.1186/1471-2164-11-553)
Supplement: Additional file 2 — Interactive Direct Interaction Network representing the molecular interactions between zinc, copper, iron, calcium and proteins encoded by transcripts changed by zinc supplementation. Mini web-site containing index.html and hyperlinked pages in subdirectory describing a Direct Interaction Network automatically generated based on curated interactions contained within the proprietary PathwayArchitect database. Ovals represent proteins and the circles symbolize metal ions. Objects are coloured by their abundance in zebrafish at the time-point they were significantly different from the control is a scale from -4 fold (dark green) to +4 fold (dark red). Where significant differences were found at more than one time-point, the colour overlay shows expression at the first instance. Dark blue squares denote 'binding', and light blue squares 'expression'; green squares stand for 'regulation', green diamonds for 'metabolism', and green circles for 'promoter binding'. Arrow heads indicate directionality of the interaction where annotated. All nodes and edges can be further interrogated by selecting the relative area of the image. [file 1471-2164-11-553-S2.zip › PathwayArchitect Zn xs DIN/864606.html]

# BINDING:

|  |  |
| --- | --- |
| Type | BINDING |
| Effect | None |


---

|  |  |
| --- | --- |
| Score | 0 |


---

|  |  |
| --- | --- |
| Reference Count | 9 |


---

|  |  |
| --- | --- |
| Mechanism | Unknown |


---

|  |  |
| --- | --- |
| Reference:0 || Sentence | "Because anthracycline cardiotoxicity is attributed to their ability to avidly bind iron (Fe), we examined the effect of anthracyclines on intracellular Fe trafficking in neoplastic cells and differentiated cardiomyocytes." |
| PMID | 12644586 |
| Year | 2003 |
| Species | Human |
| Journal | Mol Pharmacol |
| RefScore | 1 |
| Source | PArchNLP |
  |
|  |  |


---

|  |  |
| --- | --- |
 Reference:1 || Sentence | "METHODS: Hemoglobin concentration (HGB), MCV, RDW, serum iron concentration (Fe), total iron binding capacity (TIBC) and transferrin saturation percentage (TS%) were simultaneously assayed in 605 pregnant women at prenatal examination." |
| PMID | 9596875 |
| Year | 1997 |
| Species | Human |
| Journal | Zhonghua Fu Chan Ke Za Zhi |
| RefScore | 2 |
| Source | PArchNLP |
  ||  |  |


---

|  |  |
| --- | --- |
 Reference:2 || Sentence | "Serum Fe and total iron binding capacity (TIBC) were determined prior to administration of the tablets and one, two, four, and six hours after ingestion." |
| PMID | 1454637 |
| Year | 1992 |
| Species | Human |
| Journal | Pediatr Emerg Care |
| RefScore | 0 |
| Source | PArchNLP |
  ||  |  |


---

|  |  |
| --- | --- |
 Reference:3 || Sentence | "Iron (Fe) status and Fe supplementation were assessed in 20 children with phenylketonuria (PKU) through dietary intake and through measurements of ferritin, hematocrit, hemoglobin, mean cellular hemoglobin, mean cellular volume, serum Fe, total iron binding capacity, unbounded iron binding capacity, transferrin saturation and transferrin." |
| PMID | 8263269 |
| Year | 1993 |
| Species | Human |
| Journal | J Am Coll Nutr |
| RefScore | 1 |
| Source | PArchNLP |
  ||  |  |


---

|  |  |
| --- | --- |
 Reference:4 || Sentence | "Although there was no significant (p greater than 0.05) correlation between Haemoglobin (Hb), Packed cell volume (PCV), Plasma Fe, and Total Iron Binding capacity (TIBC), and erythrocyte Na+ or K+ levels during iron deficiency, analysis of variance showed that Hb, PCV, Plasma Fe and TIBC in concert significantly (p less than 0.05) affected erythrocyte Na+ in iron-deficient rats and humans, whereas K+ was significantly (p less than 0.05) affected only in rats." |
| PMID | 2535342 |
| Year | 1989 |
| Species | Human |
|  | Rat |
| Journal | J Trace Elem Electrolytes Health Dis |
| RefScore | 1 |
| Source | PArchNLP |
  ||  |  |


---

|  |  |
| --- | --- |
 Reference:5 || Sentence | "It is postulated that Fe++ binding, particularly by premicellar bile salts, may play an important physiologic role in increasing iron solubility within the intestinal lumen, thus increasing iron absorption." |
| PMID | 2376701 |
| Year | 1990 |
| Species | Human |
| Journal | J Lab Clin Med |
| RefScore | 0 |
| Source | PArchNLP |
  ||  |  |


---

|  |  |
| --- | --- |
 Reference:6 || Sentence | "The binding of iron (Fe) to human serum transferrin (Tf) was analyzed with an HPLC system equipped with an anion exchange column and directly connected with a high-resolution inductively coupled plasma mass spectrometer for metal detection." |
| PMID | 11042382 |
| Year | 2000 |
| Species | Human |
| Journal | Biochim Biophys Acta |
| RefScore | 1 |
| Source | PArchNLP |
  ||  |  |


---

|  |  |
| --- | --- |
 Reference:7 || Sentence | "The performance of a capillary magnetophoretic device was improved by enhancing the magnetic field gradient using a pair of small iron tips attached to the Nd-Fe-B magnets." |
| PMID | 12186388 |
| Year | 2002 |
| Species | Human |
| Journal | J Chromatogr A |
| RefScore | 0 |
| Source | PArchNLP |
  ||  |  |


---

|  |  |
| --- | --- |
 Reference:8 || Sentence | "Spectrophotometric titration revealed that TPP could stoichiometrically bind ferric iron to form a redox-inactive Fe-TPP complex." |
| PMID | 9374417 |
| Year | 1997 |
| Species | Human |
| Journal | Biochem Pharmacol |
| RefScore | 0 |
| Source | PArchNLP |
  |


---

|  |  |
| --- | --- |
